# Can Diet Influence Our Health by Altering Intestinal Microbiota-Derived Fecal Metabolites?

**DOI:** 10.1128/mSystems.00187-17

**Published:** 2018-03-20

**Authors:** Qiang Lyu, Cheng-Chih Hsu

**Affiliations:** a Department of Chemistry, National Taiwan University, Taipei, Taiwan

**Keywords:** GNPS, gut microbiome, liquid chromatography mass spectrometry, metabolome

## Abstract

The human gastrointestinal tract harbors a diverse, highly mutualistic microbial flora which could produce a myriad of specialized metabolites. These specialized metabolites are the chemical cellphones that gut microflora use to communicate with their human host and could potentially be used to cure diseases. Chemical compounds in diet also shape the gut flora. In order to understand which and how the gut microbe-derived specialized metabolites affect human health, the “gut microbiome-metabolomic-human health axis” is thus proposed. In our laboratory, a strategy combining genomic, chemical, phenotypical analyses has been implemented to mine the treasures of bioactive molecules found in our gut and stool. We believe that the cutting edge metabolomics will bridge microbiology and human health.

## Abstract (precis)

The human gastrointestinal tract harbors a diverse, highly mutualistic microbial flora which could produce a myriad of specialized metabolites. These specialized metabolites are the chemical cellphones that gut microflora use to communicate with their human host and could potentially be used to cure diseases.

## PERSPECTIVE

The human gastrointestinal tract harbors a diverse, highly mutualistic microbial flora, which is collectively referred to as the gut microbiome. It has been estimated that about 0.2 kg of the human body weight is constituted by these inhabitants of the gut (1). The genetic diversity of the gut microbiome is even more astonishing. Metagenomic sequencing of fecal samples has identified 3.3 × 10^6^ nonredundant microbial genes from up to 1,150 different species, outnumbering human protein-coding genes by about 150-fold. In addition, each person is estimated to host at least 160 different species (2).

Gut microbiota plays a key role in maintaining human health, and aberrant gut flora expression is involved in various diseases. Recently, more and more research has indicated that diseases such as diabetes, obesity, cancer, inflammatory bowel disease, and even Alzheimer’s disease are associated with the alterations of the microbiome. For example, an obvious gut microbial dysbiosis was observed in patients with type 2 diabetes, in which the abundance of butyrate-producing bacteria was decreased, while that of some opportunistic pathogens and microbiota involved in sulfate reduction and oxidative stress resistance was increased (3). On the other hand, after fecal microbiota transplant, gut microbial diversity of patients infected by *Clostridium difficile* recovered to the level in healthy donors, with an increase in *Bacteroidetes* species and *Clostridium* clusters IV and XIVa and a decrease in *Proteobacteria* species. The infusion of donor stools was even more effective than the use of vancomycin for the treatment of recurrent *C. difficile* infection (4). Research in the past few decades indicated that the effect of gut microbiota on human health is far more complicated than what we originally thought. It has been hypothesized that all diseases may induce a perturbation of the healthy microbiome into a diseased pathobiome, regardless of whether the disease is acute or chronic, infectious or noninfectious, and regional or systemic (5).

The study of the microbiome has become a burgeoning branch in biological sciences. In 2007, only 14 NIH grants contained the word “microbiome” in their titles or abstracts, and the number went up to 1,043 in 2017 (http://grantome.com/search?q=microbiome). This highlights how much academic attention and public interest is directed to the roles of microorganisms in our intestinal tracts, but very little of the knowledge is on how gut microbe-derived specialized metabolites affect human health. This is surprising because natural microbial products have been a rich source of new drugs over the past decades. In order to gain a more insightful understanding into host-microbe interactions, a paradigm shift of focus from the changes in intestinal flora genomics to the differences in their chemical products is required.

## GUT MICROBIOTA AND DIET

Diet is important in maintaining human health, partly by modulating the gut microbiome. Recently, both the long-term and short-term effects of diet on gut microbiome were discussed. A long-term diet of high fiber and carbohydrate could increase the level of *Prevotella*, while a typical “Western” diet of high protein and fat could increase the level of *Bacteroides* (6). Another study reported that short-term consumption of diets could rapidly induce changes in the gut microbiome, and food-borne microbes such as bacteria, fungi, and even viruses from food would colonize the gut transiently (7). Obviously, diet can be used as an effective way to manipulate the gut microbiota.

In order to illustrate how diet alters and modulates the microbial community, researchers have been making efforts to purify the main chemical compositions from many kinds of food to evaluate their effect on gut microbiome, most of them focused on polysaccharides, fiber, lipids, and proteins. On the other hand, fruits have been one of the major food sources that are rich in secondary metabolites. Phenolic compounds are one of the main classes of secondary metabolites in plants, and they have been reported to have multiple bioactivities, such as antioxidant, anticarcinogenic, antidiabetic, and antiobesity activity. However, the bioavailability of phenolic compounds has been controversial due to their low absorption rate in the gut (8). Indeed, the level of phenolic compounds in the circulatory system is as low as nanomoles per liter, and most phenolic compounds are found in the intestinal tract (9). Recently, polyphenol-rich extracts from cranberries were shown to reduce diet-induced obesity and improve insulin resistance, and notably, an increase of *Akkermansia* spp. in the gut microbiota of mice was observed (10). This gave us an insight that the gut microbiota may play a critical role in the bioactivity of phenolic compounds in diet and encouraged us to further explore the possible metabolic and bioactive mechanisms of phenolic compounds and many other compounds in diet.

**FIG 1:**
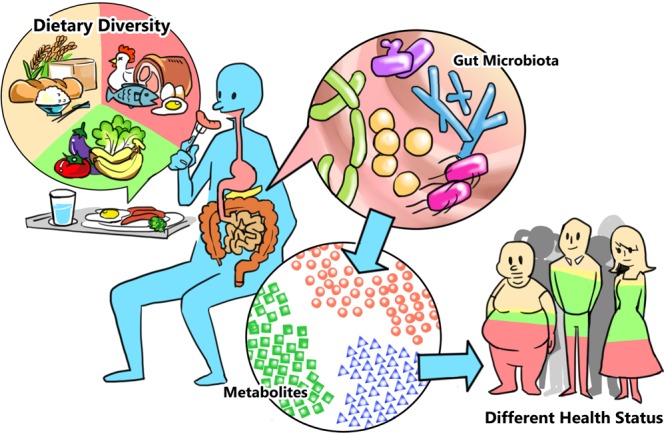
The gut microbiome-metabolomic-human health axis.

In our laboratory, we are attempting to develop multidisciplinary strategies to sketch the “gut microbiome-metabolomic-human health axis” (Fig. 1). As we hypothesize in Fig. 1, diet could alter the composition of gut microbiota, which would shape the microbial metabolome subsequently, and further influence the homeostasis of the host human body. Using bioanalytical and biochemical tools, we seek to reveal the treasure a few inches underneath our belly. We will first explore how phenolic compounds (inducer) in plants alter the consortium of the specialized metabolites produced by the gut microbiota.

## MICROBIAL SPECIALIZED METABOLITES

The gut microbiota could produce a myriad of small molecules through metabolism, including metabolites biochemically modified by microorganisms which originated from dietary components and metabolites that are synthesized *de novo* by gut microbes. It has been reported that about 10% of the metabolites in blood are associated with gut microbiota (11). In an ecological system, members of the microbiota communicate with each other and with their environmental partners/foes, largely relying on their specialized metabolites as the signaling molecules. The gut microbiota has evolved with the human body and our ancestors for millions of years, and gut microbiota may have learned to interact and communicate with us using their chemical cellphones.

Recently, several metabolites with bioactivity associated with gut microbiota were discovered. For example, the ferrichrome isolated from *Lactobacillus casei* ATCC 334 was demonstrated to have a strong tumor-suppressive effect on colon cancer cells (12). Another study indicated that α-galactosylceramide, a sphingolipid compound isolated from the membrane of *Bacteroides fragilis* NCTC 9343, might influence host immune homeostasis (13). More and more attention has shifted to the specialized metabolites produced by the gut microbiota. More importantly, as the specialized metabolites from the normal gut flora have coevolved with the human body for millions of years, our body might well have accepted them, and the side effects of using these compounds may possibly be low.

Mining the gut microflora is just like treasure hunting. Meanwhile, it is almost impossible to obtain the identities of all of the hundreds or thousands of small molecules in the gut, not to mention their highly diverse chemical structures, bioactivities, and synthetic pathways. Notably, probing biosynthetic gene clusters (BCGs) has been proved to be a powerful approach in the exploration of bioactive gut microbial metabolites, as more than 3,000 BCGs has been identified in the genomes of human-associated bacteria (14). Whether these compounds are authentically expressed in the gut is not known, and this task would be enormously labor-intensive in terms of purification, structural elucidation, and bioactivity testing. In this regard, a more systematic and efficient approach to characterize microbe-derived metabolites in a high-throughput manner is urgently needed.

## IDENTIFYING UNIQUE MICROBIOTA-ASSOCIATED MOLECULES

Several strategies have been developed to predict hypothetical metabolic products of microorganisms, but most of these strategies were based on genomic analysis. Usually, these approaches give us coarse pieces of information, instead of detailed predictions to specific molecular products, such as molecular weights and chemical structures (15). Alternatively, liquid chromatography combined with tandem mass spectrometry (LC-MS/MS) has been largely used as an efficient analytical technique in proteomics and metabolomics, in which the identities of hundreds to thousands of biological molecules could be revealed. However, such a strategy could still be inefficient in characterizing natural products, especially the specialized metabolome of gut flora, for which the database has yet to be established. Another challenge is how to differentiate the unique microbial products from endogenous host metabolites, which could be many orders of magnitude higher than the concentrations of the microbial target compounds.

**FIG 2:**
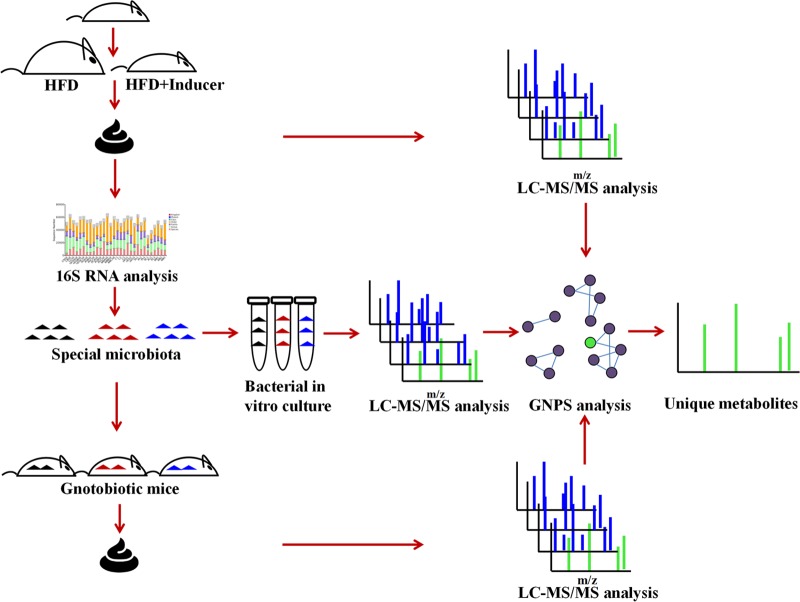
Experimental flow chart to characterize unique metabolites.

To resolve gut microbial products in greater detail, GNPS (global natural product social molecular networking) may be a solution. In this online mass spectrometry-based platform, structurally similar metabolites could be categorized in groups based on their tandem mass spectral similarity (16). In this way, thousands of molecules from food, intestinal contents, host cells, and even bacterial cultures could be systematically compared and classified based on their structural similarities. More importantly, unique microbial metabolites of a specific gut microbial species that is buried deep inside the tremendous “nonmicrobial background” and the specialized metabolome from all the other hundred microbial species could be readily recognized using GNPS (Fig. 2). In our lab, a systematic and universal methodology for mining the specialized metabolome of gut flora is implemented as illustrated in Fig. 2. We also compared the results of 16S RNA analysis of fecal samples from different groups of mice fed a high-fat diet (HFD) and chemical extracts of other food sources (as inducer) for microbiome features. The subsequent metabolome in each group of fecal samples as well as isolated gut microbial cultures are then visualized using GNPS.

## CONCLUDING REMARKS AND PERSPECTIVES

Multiple technologies such as 16S RNA and metagenomic sequencing yield a deeper understanding of how important the dynamic microbial consortia are to human health, but little is known about the metabolites derived from or modified by the gut microbiota. How does the gut flora metabolome change with the perturbation of intestinal flora? Is metabolome diversity as important as microbial diversity to human health? Gut microbiota produce a wide range of metabolites that talk to us and that coevolved with the human body for millions of years. Now is the time to translate and to address these questions. Combining multiscale data set, including genomic, chemical, and phenotypical analysis, the treasure map of molecules will then be revealed, leading us to explore the new continent of gut microbial metabolome.
